# Casting a wider net: Immunosurveillance by nonclassical MHC molecules

**DOI:** 10.1371/journal.ppat.1007567

**Published:** 2019-02-21

**Authors:** M. Patricia D’Souza, Erin Adams, John D. Altman, Michael E. Birnbaum, Cesar Boggiano, Giulia Casorati, Yueh-hsiu Chien, Anthony Conley, Sidonia Barbara Guiomar Eckle, Klaus Früh, Timothy Gondré-Lewis, Namir Hassan, Huang Huang, Lakshmi Jayashankar, Anne G. Kasmar, Nina Kunwar, Judith Lavelle, David M. Lewinsohn, Branch Moody, Louis Picker, Lakshmi Ramachandra, Nilabh Shastri, Peter Parham, Andrew J. McMichael, Jonathan W. Yewdell

**Affiliations:** 1 Division of AIDS, NIAID, Bethesda, Maryland, United States of America; 2 University of Chicago, Chicago, Illinois, United States of America; 3 Emory University, Atlanta, Georgia, United States of America; 4 Massachusetts Institute of Technology, Cambridge, Massachusetts, United States of America; 5 San Raffaele Scientific Institute, Milano, Italy; 6 Stanford University, Stanford, California, United States of America; 7 Peter Doherty Institute for Infection and Immunity, University of Melbourne, Melbourne, Victoria, Australia; 8 Oregon Health & Science University, Portland, Oregon, United States of America; 9 Division of Allergy, Immunology and Transplantation, NIAID, Bethesda, Maryland, United States of America; 10 Immunocore Limited, Abingdon, United Kingdom; 11 Columbus Technologies, Contractor to NIAID, Bethesda, Maryland, United States of America; 12 Bill & Melinda Gates Foundation, Seattle, Washington, United States of America; 13 Officer of the Director, NIAID, Bethesda, Maryland, United States of America; 14 Brigham and Women's Hospital, Boston, Massachusetts, United States of America; 15 University of California, Berkeley, California, United States of America; 16 University of Oxford, Oxford, United Kingdom; 17 Laboratory of Viral Diseases, NIAID, Bethesda, Maryland, United States of America; University of Alberta, CANADA

## Abstract

Most studies of T lymphocytes focus on recognition of classical major histocompatibility complex (MHC) class I or II molecules presenting oligopeptides, yet there are numerous variations and exceptions of biological significance based on recognition of a wide variety of nonclassical MHC molecules. These include αβ and γδ T cells that recognize different class Ib molecules (CD1, MR-1, HLA-E, G, F, et al.) that are nearly monomorphic within a given species. Collectively, these T cells can be considered “unconventional,” in part because they recognize lipids, metabolites, and modified peptides. Unlike classical MHC-specific cells, unconventional T cells generally exhibit limited T-cell antigen receptor (TCR) repertoires and often produce innate immune cell-like rapid effector responses. Exploiting this system in new generation vaccines for human immunodeficiency virus (HIV), tuberculosis (TB), other infectious agents, and cancer was the focus of a recent workshop, “Immune Surveillance by Non-classical MHC Molecules: Improving Diversity for Antigens,” sponsored by the National Institute of Allergy and Infectious Diseases. Here, we summarize salient points presented regarding the basic immunobiology of unconventional T cells, recent advances in methodologies to measure unconventional T-cell activity in diseases, and approaches to harness their considerable clinical potential.

## Introduction

Tuberculosis (TB) [1] and HIV [2] infection kill more than 2.6 million individuals per year worldwide (refer to Table 1 for acronyms and abbreviations). Devising novel approaches to elicit effective immunity is essential to global public health, because traditional vaccine approaches have failed to prevent infection or control either disease. Experts generally agree that effective vaccines for these diseases may need to harness the remarkable abilities of T cells to detect and clear intracellular pathogens, particularly T cells that recognize nonclassical MHC molecules.

**Table 1:** Acronyms and abbreviations.

Acronym/Abbreviation	Definition
αβ T cells	alpha beta T cells
Ag	Antigen
APCs	antigen-presenting cells
BCG	Bacillus Calmette-Guérin
CMV	Cytomegalovirus
ER	endoplasmic reticulum
ERAP	endoplasmic reticulum aminopeptidase
γδ T cells	Gamma delta T cells
GEM T cells	germline encoded mycolyl specific T cells
HCMV	Human Cytomegalovirus
HIV	Human Immunodeficiency Virus
HLA	Human leukocyte antigen
MAIT cells	Mucosal associated invariant T cells
MHC	Major Histocompatibility Complex
mLPA	methyl lysophosphatidic acid
NK cells	Natural killer cells
Rh	Rhesus
RM	rhesus macaques
SIV	Simian immunodeficiency virus
TAP	Transporter associated with antigen processing
TB	Tuberculosis
Tcon	conventional CD8+ T cells
TCR	T cell antigen receptor
TLRs	Toll-like receptors

To date, only a single HIV vaccine candidate, RV144, has proven even modestly effective in preventing HIV infection. HIV vaccine candidate failures can be attributed to multiple factors—the viral replication cycle; early integration into the host genome; and the highly glycosylated and antigenically plastic nature of the envelope protein, the sole target of neutralizing antibodies that form the basis for traditional vaccination. The only available licensed vaccine against TB is Bacillus Calmette-Guérin (BCG), an *M*. *tuberculosis-*like organism, and does not confer lifelong protection against active TB.

For both TB and HIV, antigen-specific conventional CD4+ and CD8+ T cells have been major targets for candidate vaccines that have had disappointing results. The absence of known correlates of protection and surrogate biomarkers of immune responses associated with different stages of TB infection and disease has crippled clinical evaluation of the vaccine candidates. New strategies are needed to improve vaccine efficacy based on both a better understanding of the mechanisms mediating protective immunity and bacterial subversion of host immunity.

**Fig 1:**
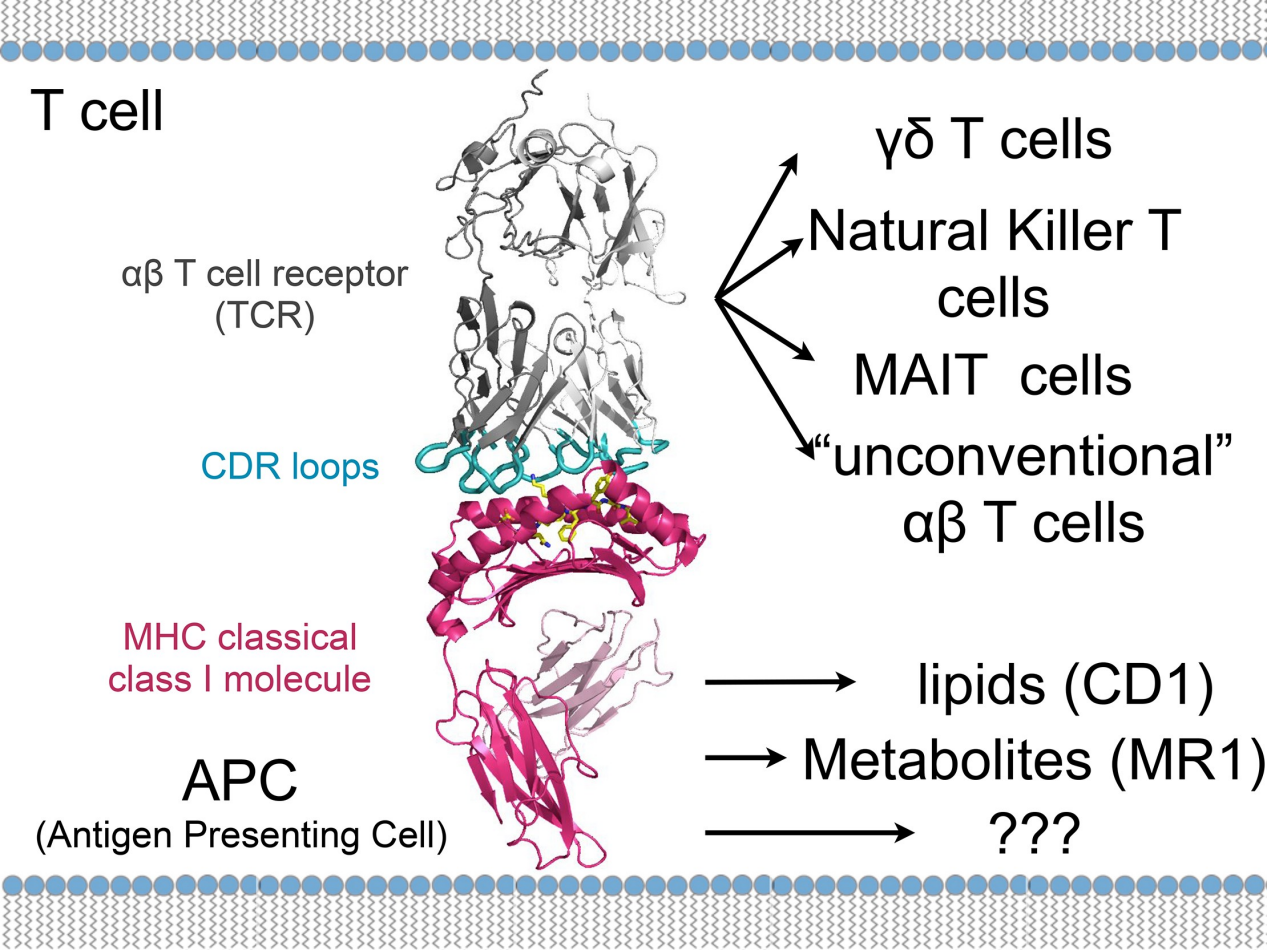
Overview of the crystal structure of the HLA-F–antigen complex. Ribbon diagrams of the extracellular portion of HLA-F in complex with β2m. The α1, α2, and α3 domains of HLA-F are in magenta. CDRs are part of the variable chains of T-cell receptors shown in cyan, where these molecules bind to their specific antigen, shown in yellow. The T-cell receptor complex with TCR-α and TCR-β chains is shown in gray. Figure provided by Dr. Erin Adams. αβ T cells, alpha beta T cells; CD1, (involved in the presentation of lipid antigens to T cells); CDR, Complementarity-determining region; γδ T cells, gamma delta T cells; HLA, human leukocyte antigen; MAIT, Mucosal associated invariant T; MHC, Major Histocompatibility Complex; MR1, major histocompatibility complex, class I-related protein; TCR, T-cell antigen receptor.

As part of the adaptive immune response, conventional cluster of differentiation (CD)4 and CD8 T cells are present in low numbers until infection or vaccination induce expansion with kinetics that vary greatly depending on the stimulus. Because conventional T cells recognize MHC class I and II molecules that display enormous genetic variability in human responses based on the generation of TCR repertoire that is itself generated by random events, conventional T-cell responses are highly variable among individuals. The principles of the classical MHC I paradigm do not accurately describe the activity of unconventional, nonclassical MHC I restricted T cells that may not recognize classical peptide antigens, are not donor restricted due to MHC polymorphism, and are present as relatively abundant populations of cells poised for rapid response—often in nonlymphoid tissues in which pathogen entry and/or replication occurs. Recent studies have shown multiple nonconventional T-cell subsets involved in protective immune responses to HIV [3] and mycobacteria [4]. Due to their utility in early defense and memory responses, these cells offer novel advantages over conventional T-cell targets in the design of anti-infectious disease strategies (see Fig 1).

A brief description of the conventional class I pathway will be useful for nonexpert readers in following the material presented below. Antigenic peptides are derived by degradation of both nascent proteins (defective ribosomal products) and “retirees,” proteins that have reached their biological life span [5]. The proteasome often plays a key role in peptide degradation, with and without the participation of ubiquitin targeting [6]. Peptides may be further trimmed by cytoplasmic amino peptidases and then transported into the endoplasmic reticulum (ER) by transporter associated with antigen processing (TAP), a dedicated transporter of peptides of less than 18 residues. Peptides are assembled in a complex consisting of TAP with dedicated and general-purpose molecular chaperones that recruit class I heavy chains and a soluble small subunit, β_2_-microglobulin (β_2_m) [7]. The last peptide amino acid trimming steps are performed by ER amino peptidases ERAP1 and ERAP2 (mice only possess ERAP1), creating a peptide of sufficient affinity to stabilize class I molecule structure and enable release from the peptide loading complex. Assembled class I molecules traverse the Golgi complex and arrive at the cell surface to enable T-cell immunosurveillance.

## Nonclassical MHC molecules

**Fig 2:**
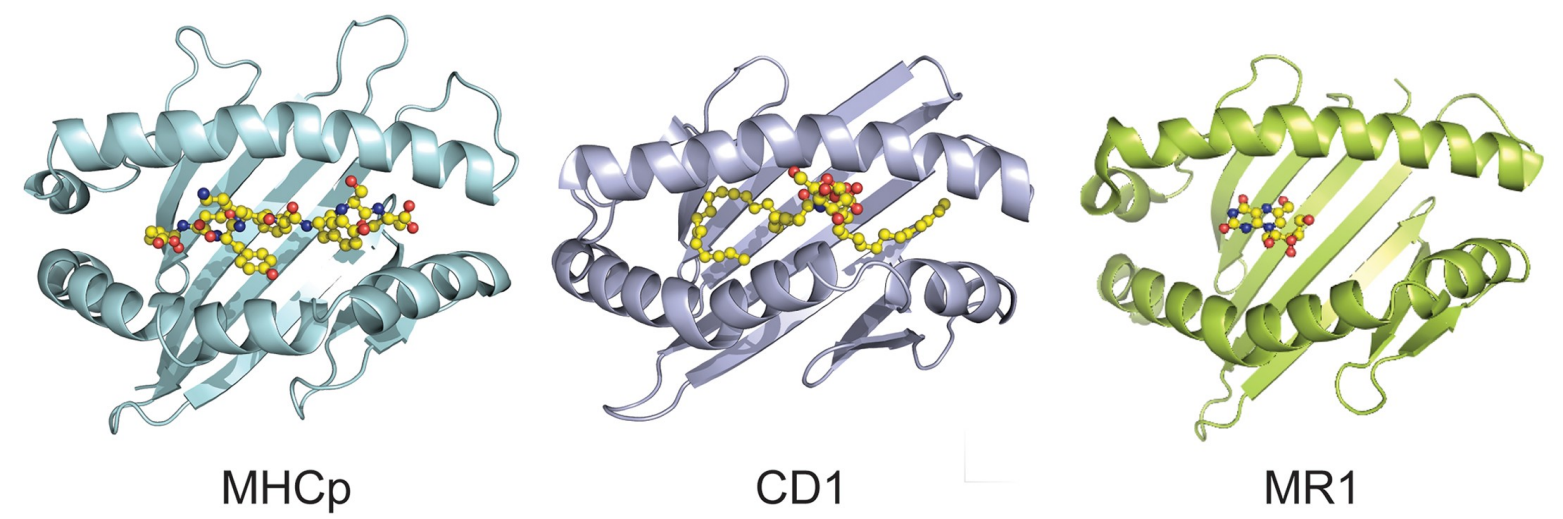
The MHC fold has evolved to present repertoires of chemically diverse antigens. Representative structures of the platform domains of classical MHC presentation of peptide antigens (H2-Kb with DEV8 peptide, PDB ID: 2CKB, on left [67]); CD1 presentation of lipid antigens (CD1d with α-Galactosylceramide, PDB ID: 1ZT4, in middle [68]); and MR1 presentation of small molecule metabolites (MR1 with 5-OP-RU, which forms a Schiff base with MR1 residue Lys43, PDB ID, on right). Figure provided by Drs. Erin Adams and Sidonia Eckle. 5-OP-RU, 5-(2-oxopropylideneamino)-6-D-ribitylaminouracil.

Humans express 18 nonclassical MHC class I and class II molecules. Despite widely disparate amino acid sequences, all family members exhibit a broadly similar structure, with a ligand binding groove at the distal end of the molecule that typically presents a small molecule (oligopeptide, lipid, or metabolite) for interaction with an immune cell receptor (see Fig 2). Nonclassical class II molecules, HLA-DM and HLA-DO, are non-peptide binding class II MHC-II homologs, that function to edit the peptides presented by MHC class II molecules. They are not known to directly recognize T cells or other immune cells. Nonclassical class I molecules include 5 encoded by MHC genes (HLA-E, F, G, MICA, and MICAB which are MHC I chain-related protein A and protein B and 11 encoded by non-MHC genes (ULBP, are a family of human cell-surface molecules distantly related to classic MHC I molecules, MR1, CD1a-e, HFE, is a protein that is similar to MHC I-type proteins and associates with beta 2-microglobulin and regulates iron absorption, FcRn, is the neonatal Fc receptor, ZAG, is zinc-α_2_-glycoprotein and EPCR is endothelial Protein C Receptor). All but HFE, FcRn, and ZAG are known to interact with either T cells or NK cells. All but EPCR, ZAG, ULBP, MIC A, and MIC B form heterodimers with β_2_-microgloubulin (β_2_m) to achieve their native structure. Eight nonclassical MHC class I molecules are currently viewed as promising targets for HIV or TB vaccines—HLA-E, HLA-F, CD1a-e, and MR1.

### HLA-E

HLA-E (with functional homologs Mamu-E in rhesus macaques and Qa-1 in mice) is expressed by nearly every nucleated cell in the body, with relatively high expression in immune system cells [8, 9]. Two alleles, differing at a single position (residue 107), are present at nearly equal frequencies in human populations. The structure of MHC-E is similar to classical MHC-Ia molecules. Under normal circumstances, however, MHC-E molecules are nearly exclusively loaded with a conserved 9-mer peptide derived from the leader sequence of HLA-A, -B, -C, or -G molecules [10]. HLA-E, bearing this peptide, down-regulates NK cell activation by interacting with CD94/NKG2A (a family of C-type lectin receptors which are expressed predominantly on the surface of NK cells) receptors [11, 12]. Counterintuitively, the processing of the signal peptide presented by HLA-E entails both proteasome processing and TAP transport [13]. Indeed, under conditions of TAP inhibition, which occurs naturally during viral infections that express TAP inhibitors, the HLA-E peptide repertoire increases massively, potentially enabling T-cell immunosurveillance [14]. In mice, a TAP blockade elicits an alternate, MHC-E (Qa-1 which is the murine equivalent restricted CD8+ T-cell response that protects mice against tumors [15]. HLA-E restricted CD8+ T cells have been detected in humans, suggesting this cellular subset could potentially mediate protection against cells bearing antigen processing machinery defects, such as in neoplasms.

The mechanism by which Qa-1 functions to monitor cellular endoplasmic reticulum aminopeptidase (ERAP) activity, an ER resident protein, which plays a key step in the MHC I antigen processing pathway in performing the final cuts in peptide ligand processing was detailed by Shastri. Cells from ERAP knockout mice elicit a strong CD8+ T-cell response from wild-type mice specific for Qa-1 bound to a peptide from highly conserved host proteins (Fam49a and Fam49b). This peptide is only presented in cells lacking ERAP activity. Qa-1–specific T cells are present in nonimmunized mice in relatively large numbers and with memory markers, suggesting their constitutive activation [16]. They might play a critical role in immunity to herpesviruses, which are known to down-regulate ERAP [17] but likely have additional function as well, because laboratory mice are generally free of herpesvirus infections.

Demonstrating the surprising potential of this nonclassical system in classical infection scenarios, Picker provided an update on progress from his team in developing HIV and TB vaccine vectors based on the β-herpesvirus, cytomegalovirus (CMV). The approach exploits the unique immunobiology of CMV, which induces durable, high frequency, effector-differentiated, circulating and resident CD4+ and CD8+ T-cell responses to many CMV gene products and likely extends to inserted vaccine immunogens. They have shown that approximately 50% of rhesus macaques (RM) vaccinated with strain 68–1 Rhesus (Rh) CMV vectors expressing SIV (Simian immunodeficiency virus) Ag inserts manifest immediate control and eventual clearance of mucosally administered, highly pathogenic SIVmac239 [18, 19]. Similarly, RhCMV vector-expressing TB Ag inserts show nearly 70% efficacy in disease reduction after intrabronchial challenge with Erdman strain TB, including 40% of RMs that were completely protected against disease [4].

Detailed investigation of the immunologic properties of the RhCMV vector used in these studies revealed remarkable unconventional CD8+ T-cell responses that exclusively target peptides presented by MHC-II and MHC-E. This highly unconventional response requires deleting several RhCMV genes [3, 20]. Replacing the affected genes—specifically, two genes that encode proteins which form the pentameric receptor complex—reverted the CD8+ T cell response to recognize MHC-Ia. Although the ability of RhCMV/TB vectors to protect against TB challenge was maintained with this gene-repair and response reversion, the efficacy of RhCMV/SIV vectors against SIV challenge was abrogated, suggesting the unconventionally restricted CD8+ T cells play a critical role in the ability of RhCMV/SIV vector-elicited responses to control SIV challenge. The functions of these novel T-cell populations in pathogen control can now be addressed in various infectious disease models.

### HLA-F

The function of HLA-F remains largely enigmatic. By interacting with NK cell receptors, HLA-F is thought to function in maternal-fetal tolerance and possibly in infection and autoimmunity. Adams described how, unusually for a class I molecule, which typically requires a peptide to stabilize its dimeric structure (heavy chain plus β_2_m), that HLA-F can exist as a heavy-chain only (without β_2_m) “open-conformer” [21]. Such open conformers interact with distinct NK receptors to form liganded HLA-F molecules [22–25], indicating that the molecule exists in two different native states—with and without β_2_m [26].

Because residue 62 in HLA-F is tryptophan, in contrast to arginine in other class I molecules, N-termini of peptide ligands cannot bind to the altered A pocket, and most peptides extend from the binding groove [26]. Therefore, peptides recovered from HLA-F exhibit a length distribution typical of class II bound peptides. This modification is specific for humans and orangutans among all primates. This difference may contribute to variances between monkey and human immunity to infectious diseases.

### CD1 family of molecules and presentation of lipids

CD1 proteins are a family of evolutionarily conserved class I molecules that present lipids for immunosurveillance. The five human CD1 isoforms exhibit distinct cellular expression profiles, lipid-ligand repertoires, and functions. Moody described how mass spectrometry has identified numerous TB and other bacterial lipid CD1 ligands, including mycolyl lipids, sulfolipids, mycoketides, and phospholipids [27]. These antigens have been used to study cellular mechanisms of lipid loading onto CD1 proteins in dendritic cells and the roles of Toll-like receptors (TLRs) in promoting cellular antigen presentation [28]. CD1b is essentially monomorphic in the human population, meaning that unlike vaccines targeting classical class I molecules, CD1b-targeted vaccines have potential for universal use among genetically unrelated human subjects. Another potential advantage of CD1-based vaccines is that, unlike peptides, lipids are immutable, so escape would require mechanisms that alter lipid generation or trafficking.

A striking characteristic of many CD1-restricted T cells is autoreactivity against different types of antigen-presenting cells (APCs) in the absence of foreign antigens, implying that they recognize self-lipid molecules. Recent studies have identified lipid autoantigens present in mammalian cell membranes, including common sphingolipids and phospholipids [29]. Studies from the Moody laboratory have identified new populations of human T cells, including interleukin-22 (IL-22 secreting CD1a autoreactive T cells [29] and germline encoded mycolyl-specific (GEM) T cells [30]. The GEM TCR docks centrally above CD1b, whereby the conserved TCR α-chain extensively contacts CD1b and can activate conserved populations of responding T cells. These cells are “donor-unrestricted” and show TCR gene usage patterns more broadly conserved across human populations [31]. Ultimately, a basic understanding of the cellular mechanisms that allow T cells to discriminate among self- and foreign antigens can be leveraged to formulate lipid antigens and adjuvants as therapeutic immunomodulatory agents and vaccines. More generally, how CD1-restricted self-reactivity impacts T-cell activation and its potential for tumor destruction versus autoimmunity must be assessed.

Indeed, Casorati described a novel self-lipid antigen, methyl lysophosphatidic acid (mLPA), overexpressed in malignant cells that stimulates CD1c self-reactive T cells. Cytotoxic mLPA specific-T cells control leukemia growth in vitro and in vivo. These findings point to CD1c and self-lipids as new potential targets for leukemia immunotherapy [32]. Difficulties in isolating these cells from peripheral blood can be bypassed by cloning the mLPA-specific TCR into a lentiviral vector to engineer T cells before their expansion and subsequent transfusion into leukemia patients. An important tool for obtaining basic understanding of CD1c-based therapy is the CD1c transgenic mouse (CD1c is absent in mice), which can be combined with retrogenic T cells [33].

## Immune cells recognizing class Ib molecules

### γδ T cells

Gamma delta (γδ) T cells, like alpha beta (αβ) T cells, use somatic V, D, J gene rearrangement to generate diversity in antigen receptor specificity [34]. Although γδ T cells and αβ T cells exert similar effector functions, these subsets differ in antigen recognition and activation requirements. Although classical and nonclassical MHC molecules can be recognized by γδ T cells, they are not obligatory components of γδ T-cell antigens. Indeed, γδ TCRs have antibody-like antigen recognition properties, and γδ T cells can recognize common B-cell antigens like phycoerythrin and haptens [34, 35].

γδ T cells are the major initial source of IL-17 production in mouse models of infection and autoimmune diseases, acting within hours of infectious challenge, likely triggered by very early cytokine responses [36, 37]. Human γδ T cells respond to microbial metabolites and transformed cells, suggesting their participation in anti-infection and antitumor immunity [38, 39]. Chien reported that hapten specific-γδ T cells can recognize metabolites, post-translational modifications, peptides, and proteins of both host and foreign origin, indicating that γδ TCRs can generate broadly reactive TCRs. This recognition suggests that γδ T cells increase TCR degeneracy to maximize the coverage of their TCR repertoire. How this impacts their self-reactivity is an important future question.

### Mucosal associated invariant T cells

Mucosal associated invariant T (MAIT) cells recognize the monomorphic MHC-I like molecule MR-1, which presents antigens that originate from a metabolic intermediate in the microbial biosynthesis of riboflavin [40]. MR-1 also binds folate-derived nonstimulating ligands, which act as competitive inhibitors of immunogenic ligands [41]. Eckle described how the screening of 7,000 small molecules led to 20 compounds, representing 8 structural classes, which impacted MAIT-cell activation. Therefore, MR-1 can accommodate a range of chemical structures, consistent with the existence of additional natural MR-1 ligands that activate MAIT and possibly other T cells [42].

A mouse model for Legionnaires disease revealed that MAIT cells accumulate in the lungs of mice infected with legionella bacteria. MAIT-cell–deficient mice display decreased bacterial clearance. MAIT cell numbers remain elevated for at least 300 days postinfection, consistent with exerting a memory function. Protection by MAIT cells was enhanced in immunodeficient RAG2^-/-^γC^-/-^ mice, in which lethality was averted by the transfer of MAIT cells. The use of adoptive transfer of in vivo expanded MAIT cells provides compelling evidence that MAIT cells can confer protection against important human pathogens and demonstrates that this protection depends upon their capacity to produce interferon gamma (IFN-γ) [43].

### Potential for T-cell–based vaccines

Mtb resides in immune-cell phagosomes. How TB peptides are processed and presented on MHC class I molecules is a topic of great interest. Lewinsohn used HLA-Ia–restricted T cells to define epitopes displayed by HLA-B, which appear to dominate the response [44–46]. Presentation of many peptides is dependent on both TAP and proteasomes. As both TAP and HLA-Ia molecules are present in the phagosome with proteasomes coating the cytoplasmic surface, presentation seems to entail export of Mtb proteins and/or peptides to phagosome bound proteasomes whose peptide products are reimported into the phagosome by TAP. This mechanism appears to also account for Mtb peptide presentation on HLA-E, which is also TAP and proteasome dependent [47, 48]. By contrast, Mtb metabolite presentation on MR-1 was TAP and proteasome independent, as expected [49].

Lewinsohn also described a chimeric MR-1 molecule developed to purify Mtb-derived ligands from infected macrophages [50]. For a number of peptides that were identified by mass spectrometry, immunogenicity was confirmed using T-cell clones. Although MAIT cells previously were believed to use a limited set of TCRs, T cells recognizing these ligands used highly diversified TCRs. These unexpected discoveries underscore that further research is needed to understand basic MAIT cell physiology and how it may be leveraged to treat TB and other microbial diseases.

Clearly much remains to be learned about MAIT cells and how they might be manipulated to treat TB and other microbial diseases.

## Technologies to measure nonconventional T-cell responses

Understanding and manipulating nonconventional T cells requires identifying the activating ligands bound to class Ib molecules and delineating the TCR repertoire elicited. In many cases, this requires the development of novel technologies.

### Defining the HLA-E immunopeptidome

The protection conferred by strain 68–1 RhCMV/SIV-induced SIV-specific CD8+ T cells in RM has resurrected T-cell vaccine development [18, 51]. Because this remarkable mechanism and efficacy of protection has not been observed with other T-cell–based vaccines for SIV/HIV, translating these preclinical results into a human vaccine candidate is a major priority. At the molecular level, considerable effort has been expended to understand the highly unconventional CD8+ T-cell responses, including the extent to which they depend on the unique MHC complexity present in RM—which can possess more than 20 highly diverse class Ia genes—and the astonishingly fiendish mechanisms utilized by CMV [52] to modulate class I presentation and T-cell activation.

Previous work from the McMichael laboratory revealed that HLA-E, like Qa-1, nearly homogeneously presents an HLA class I signal peptide (termed VL9) to inhibitory NKG2/CD94 NK-cell receptors [11]. Human CMV (HCMV) subverts this system by expressing VL9 in the leader sequence of its UL40 protein, thus preventing NK detection of HCMV down-regulation of classical class I molecules. Liberation of VL9 from UL40 is independent of TAP-mediated peptide transport into the ER and ERAAP activity in the ER [53]. This enables CMV to shut down these important peptide-generating pathways (which are required for VL9 presentation from host class I molecules) and still present its own VL9 on HLA-E in virus-infected cells [54]. In cells infected with RhCMV 68–1, Mamu-E presents hundreds of virus-encoded peptides, including peptides from non-CMV viral genes inserted into the vector. Because T cells induced by these vectors recognize SIV infected cells, this indicates that Mamu-E can present SIV encoded peptides at sufficient levels to enable immunosurveillance of SIV-infected cells [3, 20].

McMichael described the crystal structures of HLA-E in complex with HIV and Mtb-derived peptides. Primary HLA-E anchor-binding residues are largely conserved among VL9 peptides. Pathogen-derived peptide binding to HLA-E is measured using a combined sandwich enzyme-linked immunosorbent assay (ELISA) or single-chain trimer approach. These analyses broaden binding capacity of the primary peptide binding pockets, originally reported for HLA-E-binding peptides [55]. Several HIV epitopes identified in RhCMV68-1 HIV-1 Gag-insert vaccine trials, including some that lacked canonical anchor residues, bind to HLA-E, albeit with considerably lower affinity than VL9. Similarly, screens of previously reported HLA-E restricted microbial peptides, including an Mtb-derived panel, identified peptides, including a Mtb peptide that binds with similar affinity to VL9. HLA-E and β2m fold in the absence of added peptide, suggesting that HLA-E is relatively stable without peptide, thus favoring both low-affinity peptide binding and consequent peptide exchange. These characteristics likely favor promiscuous peptide loading in vivo, especially when the peptide loading complex is disrupted or absent in a peripheral intracellular compartment.

Altman described a potentially robust method to assess peptide binding to HLA-E based on competitive inhibition of fluorescent VL9 binding to HLA-E [56]. All peptides tested, including E-restricted epitopes that elicit T cell responses by RhCMV vaccine vectors or TB, demonstrated HLA-E binding with at least 100-fold lower affinity than VL9 itself. This raises questions about how such peptides can bind HLA-E in the ER in the presence of VL9 and how such low affinity complexes can persist on the cell surface for a sufficient period to enable immunosurveillance.

The mechanism underlying how these low-affinity peptide epitopes elicit CD8^+^ T-cell responses remains unclear. Also unknown is whether RhCMV68-1 vaccinated macaques generate unusual forms of Mamu-E resembling the HLA-E “open” conformer and whether CD8^+^ T cells recognize these forms. The properties of this RhCMV vector serendipitously revealed facets of T-cell recognition that may have gone decades, or perhaps even centuries, before being discovered.

### Single-cell technology

Leaps in next generation sequencing technology are powering investigation of the TCR repertoire and the T-cell transcriptome at the single cell level [57–59]. Huang detailed single-cell analysis of human CD8+ T cells responding to Mtb, which identifies populations of T cells expressing either CD137 or CD154 (CD40L). Single-cell TCR sequencing showed that the CD154+ population largely consisted of MAIT and NKT cells whereas the CD137+ population primarily contained conventional CD8+ T cells (Tcon) [60, 61]. Single-cell transcriptomic analyses of activated Tcon and MAIT/NKT cells detected over 2,000 messenger RNA (mRNA) species and revealed distinct population-specific signatures. For example, T cell immunoreceptor with Ig and ITIM domains (TIGIT), C-C motif ligand 3 and 4 (CCL3 and CCL4, respectively) are expressed by Tcons following activation, whereas signaling lymphocytic activation molecule family 1 (SLAMF1), TNF, IFN-γ, and C-C motif ligand 20 (CCL20) are expressed by MAIT/NKT cells following activation. A gene expression signature associated with clonal expansion was observed only in Tcons, whose proliferation was regulated by IL2RA expression.

Looking forward, single-cell technology is likely to reveal the dynamic complexity underlying the phenomenon of immunity. Advances in mass spectrometry will similarly enable single-cell proteomics, lipidomics, glycomics, and peptidomics. Ultimately, the limiting factor to developing clinical applications will not be the biomedical technology but integrating and interpreting complex data sets.

### TCR-based platforms

Recombinant TCRs offer the possibilities of directing immune responses to cells expressing nominal peptide-MHC complexes and blocking pathological immune recognition in autoimmunity in a highly specific manner. Hassan described a platform that enables the production of soluble monoclonal TCRs with enhanced affinity and specificity for targeted peptide class I complexes [62]. Immune mobilizing monoclonal TCRs against cancer are a new class of bispecific TCRs engineered to present given immune cells to cells expressing the relevant MHC-peptide complex [63]. These agents target antigens present at low epitope number with picomolar drug levels and induce durable tumor shrinkage with a favorable safety profile in malignant melanoma patients [62–65].

In the pipeline are novel molecules to target killing of CD4+ T cells infected with treatment-resistant HIV and of Mtb-infected macrophages [65]. To expand into broader-based therapy not limited by MHC polymorphism, this TCR biologic technology can be expanded to target class Ib molecules. Though the possibilities are enormous, potential off-target effects increase with broader tissue expression of targeted MHC ligand complexes.

Findings from several decades of evaluating the potential of TCRs indicate that it is possible to increase the affinities of natural TCRs through directed amino acid substitutions guided by in vitro evolution. Although the ability to accurately predict the effect of given substitutions on TCR function remain a distant goal, display library technologies have become robust, if not yet routine, for generating novel TCRs with improved features, as well as peptide ligands that can replace the bona fide nominal peptide. Birnbaum described how such libraries can be used to identify foreign peptides that induce self-peptide T-cell responses in multiple sclerosis [66], tumor specific peptides recognized by tumor infiltrating T cells, and peptide repertoires able to bind given MHC class II, class Ia, or class Ib molecules.

## Perspectives

Due largely to the history of discoveries in cellular immunology, primacy has been traditionally given to the study of classical class I molecules. Pioneers of nonclassical MHC molecule research vividly recount having to overcome skepticism that class Ib molecules are important—in part because of the circular argument that they were not widely studied.

Convening these pioneers has made it clear that not only are class Ib molecules numerically superior to class Ia molecules (16 to 3 in humans) but are also of equal biological importance. The RhCMV work has demonstrated an ability to elicit unusually broad CD8+ T-cell responses that recognize conventional and/or unconventional epitopes. The important remaining knowledge gaps regarding class Ib molecules were agreed upon:

1. What are the peptidic or nonpeptidic antigenic determinants for class Ib MHC molecules, and how do the pathways for ligand generation and loading differ from class Ia molecules?
2. What are the special features of T cells that recognize class Ib molecules?
3. How might NK and T-cell class Ib recognition be harnessed to prevent and treat infectious diseases, cancer, and autoimmunity?

The spectrum of potential nonclassical immune responses is broad and has far-reaching implications for basic and clinical immunology and for the development of vaccines that target immune-evasive pathogens, autoimmunity, or even cancer. Clinical benefit may result from the study of T cells specific for bacterial metabolic products, CD8+ T cells that recognize HLA-E or class II molecules and can potentially cure SIV or TB, and T cells that recognize specific lipids presented by CD1. A clear need persists, therefore, to increase translational research capacity, including augmenting capabilities in human immunology, virology, and microbiology, as well as developing more appropriate reagents and tools to decode these responses across species.

Class Ib MHC molecules are the allegorical poster children for the importance of maintaining an open mind in making ground breaking discoveries. As immunology textbooks expand, there is tendency to believe that the basic principles of the field are sacrosanct. The past decade of class Ib studies demonstrate this presumption is misguided. We know now, for example, that T cells are specific for bacterial metabolic products and that CD8+ T cells that can cure SIV in nonhuman primates recognize HLA-E molecules! We may even be able to treat cancer with T cells that recognize specific lipids presented by CD1.

Perhaps the paramount take-away is the importance of unfettered curiosity-based research combined with the spirit that textbooks are meant to be continuously rewritten.
